# Multi-location evaluation of mungbean (*Vigna radiata* L.) in Indian climates: Ecophenological dynamics, yield relation, and characterization of locations

**DOI:** 10.3389/fpls.2022.984912

**Published:** 2022-09-20

**Authors:** Ashok K. Parihar, Sanjeev Gupta, Kali K. Hazra, Amrit Lamichaney, Debjyoti Sen Gupta, Deepak Singh, Raju Kumar, Anil K. Singh, Rakesh Vaishnavi, M. Samuel Jaberson, Sankar P. Das, Jai Dev, Rajesh K. Yadav, B. S. Jamwal, B. R. Choudhary, O. P. Khedar, Vijay Prakash, Harsh K. Dikshit, R. K. Panwar, Manoj Katiyar, Pankaj Kumar, C. S. Mahto, H. K. Borah, M. N. Singh, Arpita Das, A. N. Patil, H. C. Nanda, Vinod Kumar, Sumer D. Rajput, D. A. Chauhan, M. H. Patel, Raja R. Kanwar, Jitendra Kumar, S. P. Mishra, Hitesh Kumar, Indu Swarup, Suma Mogali, D. Kumaresan, Narayana Manivannan, M. Byre Gowda, Muthaiyan Pandiyan, Polneni J. Rao, D. Shivani, A. M. Prusti, P. Mahadevu, K. Iyanar, Sujata Das

**Affiliations:** ^1^ICAR-Indian Institute of Pulses Research, Kanpur, India; ^2^Indian Council of Agricultural Research, Krishi Bhawan, New Delhi, India; ^3^ICAR-Indian Agricultural Statistics Research Institute, New Delhi, India; ^4^Sher-e-Kashmir University of Agricultural Sciences and Technology (SKUAST), Srinagar, India; ^5^Central Agricultural University, Imphal, India; ^6^ICAR Research Complex for North Eastern Hilly Region, Agartala, India; ^7^Chaudhary Sarwan Kumar Himachal Pradesh Krishi Vishvavidyalaya, Palampur, India; ^8^Chaudhary Charan Singh Haryana Agricultural University, Hisar, India; ^9^Pulses Research Sub-Station, SKUAST-Jammu, Srinagar, India; ^10^Agriculture University, Jodhpur, India; ^11^Rajasthan Agricultural Research Institute, Jaipur, India; ^12^Agricultural Research Station, Sriganganagar, India; ^13^Indian Agricultural Research Institute, New Delhi, India; ^14^Govind Ballabh Pant University of Agriculture and Technology, Pantnagar, India; ^15^Chandra Shekhar Azad University of Agriculture and Technology, Kanpur, India; ^16^Acharya Narendra Deva University of Agriculture and Technology, Ayodhya, India; ^17^Birsa Agricultural University, Ranchi, India; ^18^Regional Agricultural Research Station, Shillongani, India; ^19^Institute of Agricultural Science, BHU, Varanasi, India; ^20^Bidhan Chandra Krishi Viswavidyalaya, Mohanpur, India; ^21^Dr. Panjabrao Deshmukh Krishi Vidyapeeth, Pulses Research Unit, Akola, India; ^22^Indira Gandhi Krishi Vishwavidyalaya, Raipur, India; ^23^Jawaharlal Nehru Krishi Vishwa Vidyalaya, Regional Agricultural Research Station, Sagar, India; ^24^Mahatma Phule Krishi Vidyapeeth, Rahuri, India; ^25^Navsari Agricultural University, Navsari, India; ^26^Sardarkrushinagar Dantiwada Agricultural University, Sardarkrushi Nagar, India; ^27^S.G. College of Agriculture and Research Station, Jagdalpur, India; ^28^Rajmohni Devi College of Agriculture and Research Station, Ambikapur, India; ^29^Mahatma Gandhi Chitrakoot Gramodaya Vishwavidyalaya, Chitrakoot, India; ^30^Banda University of Agriculture and Technology, Banda, India; ^31^Regional Research Centre on Pulses, College of Agriculture, Indore, India; ^32^University of Agricultural Sciences (UAS), Dharwad, India; ^33^Tamil Nadu Agricultural University (TNAU), Coimbatore, India; ^34^National Pulse Research Centre (TNAU), Vamban Colony, India; ^35^University of Agricultural Sciences, Gandhi Krishi Vigyana Kendra (GKVK), Bangalore, India; ^36^Agriculture Research Station, Virinjipuram, India; ^37^Regional Agricultural Research Station (PJTSAU), Warangal, India; ^38^PJTSA-Agricultural Research Station, Madhira, India; ^39^Odisha University of Agriculture and Technology, Bhubaneswar, India; ^40^College of Agriculture, UAS, GKVK, Mandya, India

**Keywords:** crop phenology, genotype × environment (G × E) interaction, HA-GGE biplot, mega-environment analysis, adaptability

## Abstract

Crop yield varies considerably within agroecology depending on the genetic potential of crop cultivars and various edaphic and climatic variables. Understanding site-specific changes in crop yield and genotype × environment interaction are crucial and needs exceptional consideration in strategic breeding programs. Further, genotypic response to diverse agro-ecologies offers identification of strategic locations for evaluating traits of interest to strengthen and accelerate the national variety release program. In this study, multi-location field trial data have been used to investigate the impact of environmental conditions on crop phenological dynamics and their influence on the yield of mungbean in different agroecological regions of the Indian subcontinent. The present attempt is also intended to identify the strategic location(s) favoring higher yield and distinctiveness within mungbean genotypes. In the field trial, a total of 34 different mungbean genotypes were grown in 39 locations covering the north hill zone (*n* = 4), northeastern plain zone (*n* = 6), northwestern plain zone (*n* = 7), central zone (*n* = 11) and south zone (*n* = 11). The results revealed that the effect of the environment was prominent on both the phenological dynamics and productivity of the mungbean. Noticeable variations (expressed as coefficient of variation) were observed for the parameters of days to 50% flowering (13%), days to maturity (12%), reproductive period (21%), grain yield (33%), and 1000-grain weight (14%) across the environments. The genotype, environment, and genotype × environment accounted for 3.0, 54.2, and 29.7% of the total variation in mungbean yield, respectively (*p* < 0.001), suggesting an oversized significance of site-specific responses of the genotypes. Results demonstrated that a lower ambient temperature extended both flowering time and the crop period. Linear mixed model results revealed that the changes in phenological events (days to 50 % flowering, days to maturity, and reproductive period) with response to contrasting environments had no direct influence on crop yields (*p* > 0.05) for all the genotypes except PM 14-11. Results revealed that the south zone environment initiated early flowering and an extended reproductive period, thus sustaining yield with good seed size. While in low rainfall areas *viz*., Sriganganagar, New Delhi, Durgapura, and Sagar, the yield was comparatively low irrespective of genotypes. Correlation results and PCA indicated that rainfall during the crop season and relative humidity significantly and positively influenced grain yield. Hence, the present study suggests that the yield potential of mungbean is independent of crop phenological dynamics; rather, climatic variables like rainfall and relative humidity have considerable influence on yield. Further, HA-GGE biplot analysis identified Sagar, New Delhi, Sriganganagar, Durgapura, Warangal, Srinagar, Kanpur, and Mohanpur as the ideal testing environments, which demonstrated high efficiency in the selection of new genotypes with wider adaptability.

## Introduction

Mungbean (*Vigna radiata* L. Wilczek) is a vital grain legume extensively cultivated in South Asia, Africa, South America, and Australia (Pratap et al., 2015; Parihar et al., 2017a). India is the largest mungbean-producing country, accounting for about 65% of the world acreage and 54% of the production (Baraki et al., 2020). Mungbean, being a short-duration crop, is currently gaining wider recognition for diversification/intensification of cereals-based cropping systems, dietary diversification, resource conservation, and ecosystem services (Das et al., 2019; Hazra et al., 2020). Mungbean is an admirable source of digestible protein (25–28%) that offers a healthy supplement to a cereal-based diet for resource-poor vegetarian populations (Kumar et al., 2014; Parihar et al., 2017a).

Presently, the yield potential of mungbean in the Indian subcontinent is relatively low compared to other major grain legumes (Gupta et al., 2013; Kim et al., 2015; Paramesh et al., 2016; Parihar et al., 2017b). According to the physiographic and climatic conditions, the total geographical area of India is divided into 15 agro-climatic zones by the planning commission of the government of India (Ahmad et al., 2017). The diverse nature of Indian climates is well acknowledged worldwide for sustainable food production (Hinz et al., 2020). Nevertheless, climatic and edaphic variations across the country pose great difficulty in designing breeding programs. Often, the improved cultivar(s) fail to realize the potential yield due to the greater influence of different climatic and edaphic factors across the country. Therefore, understanding the crop's response to the contrasting environments would provide valuable insight into site-specific crop response and genotype × environment interaction (Elias et al., 2016). This approach will be helpful in identifying and selecting the stable, high-yielding genotypes/varieties that are best suited for a given set of environmental conditions (Islam et al., 2021) and will provide useful guidance for redesigning the present breeding approaches. Eco-physiological response and crop yield potential are often determined by crop phenological dynamics, which have a direct influence on source-sink balance (Rachaputi et al., 2019). Crop phenological events such as flowering time, total crop duration, and grain filling (reproductive) period are largely influenced by agro-climatic conditions (Raza et al., 2019; Lamichaney et al., 2021). However, the environment-induced variation of crop phenological dynamics on mungbean crops at a large scale has not been evaluated in Indian climates. Understanding the associations of crop phenological events with crop yield potential considering diverse agro-regions would provide valuable insights into site-specific crop characterization.

Crop breeding programs are mostly confined to some major agroecology, particularly at the research stations, but the promising breeding lines are grown across the country to assess their overall performance. Evaluation of mungbean breeding lines across the country is a cumbersome process, especially in identifying cultivars for the national interest. A realistic and timely approach to accelerating a variety of release programs would be to carefully select mega environments or identify strategic locations using different tactics. The multi-location trial is the prerequisite for cultivar testing and subsequent release of potential breeding lines by the All India Coordinated Research Project (AICRP) under the umbrella of the Indian Council of Agricultural Research (ICAR) for the development/testing/identification of technologies. So far, various statistical approaches have been extensively adopted, such as principal component analysis (PCA), linear regression models, additive main effects, and multiplicative interactions (AMMI), to analyze and interpret multi-location trial data (Zobel et al., 1998; Gauch, 2006; Thangavel et al., 2011; Wang et al., 2016; Baraki et al., 2020). Recently, the genotype main effect plus genotype × environment interaction (GGE) biplot is being preferred and used to portray more valid inferences as it consolidates the supremacy of additive and multiplicative models (Yan et al., 2000, 2007; Yan and Kang, 2003).

The study aimed to investigate the sensitivity of phenological events and yield traits in superior breeding lines in 39 test environments covering the diverse agroecology of the Indian subcontinent. The major objectives of the study were (i) to determine the site-specific performance of mungbean and understand G × E interaction, (ii) to evaluate the influence of environment-induced alteration in crop phenology and weather variables on mungbean productivity, and (iii) to identify strategic testing locations for target-oriented mungbean breeding programs in Indian climates.

## Materials and methods

### Study environments and weather variables

**Figure 1:**
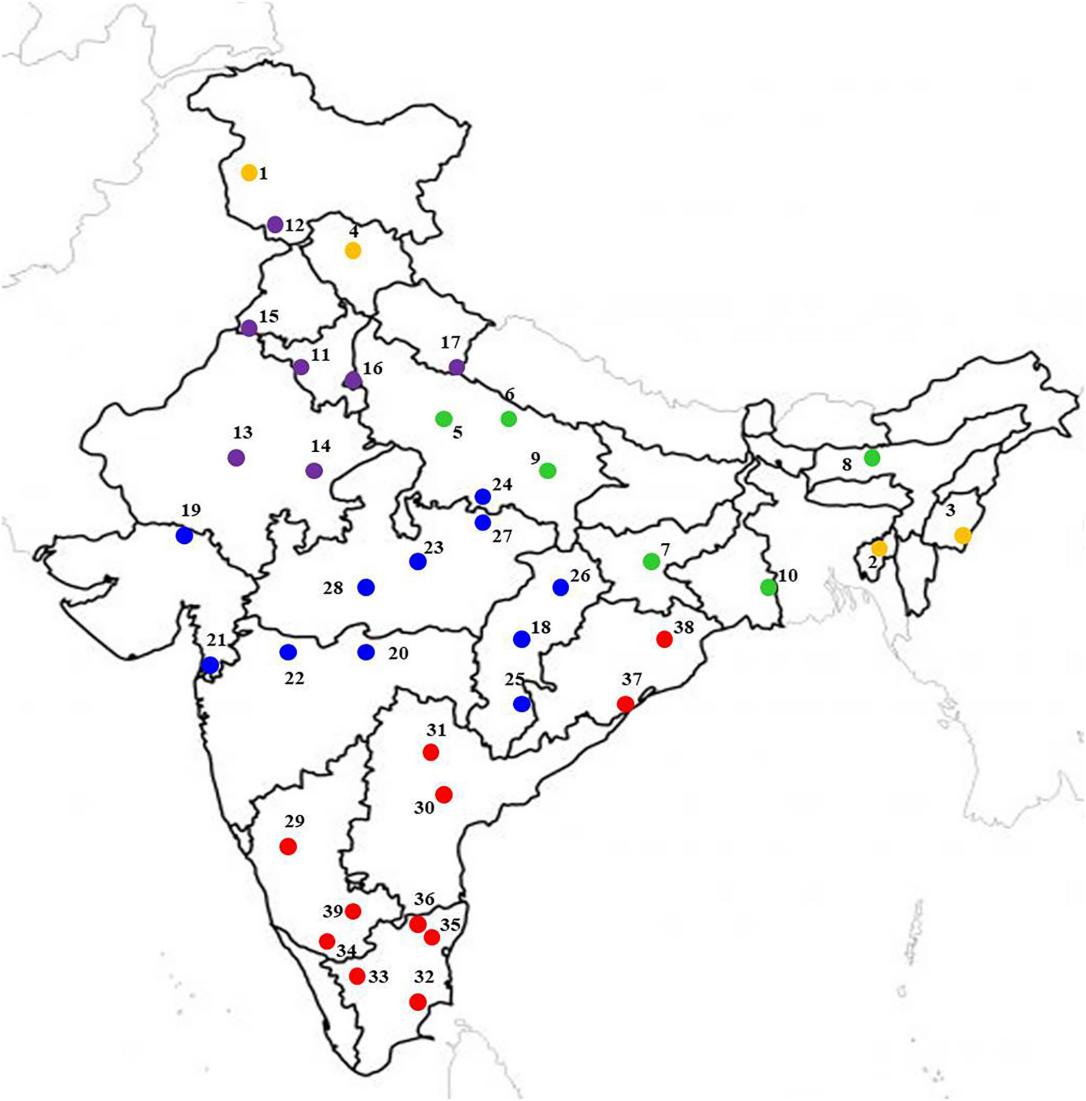
Multi-location study sites located at major mungbean producing areas in different agroecological zones of India. The yellow, green, violet, blue, and red color location dot represents North Hill Zone (NHZ), North Eastern Plain Zone (NEPZ); North Western Plain Zone (NWPZ); Central Zone (CZ), and South Zone (SZ), respectively.

The locations were representative of all possible mungbean growing ecologies of India (Figure 1), distributed throughout the length and breadth of the country with latitudes from 11.00°N at Aduthurai (Tamil Nadu) to 33.98°N at Srinagar (Jammu and Kashmir), longitude from 72.02°E at SK Nagar (Gujarat) to 93.57°E at Imphal (Manipur), and altitude from 12.0 m at Navasari (Gujarat) to 1617 m above sea level at Srinagar. The rainfall ranged from 5.3 mm at Sriganganagar (Rajasthan) to 1593.4 mm at Jagdalpur (Chhattisgarh). The maximum temperature varied between 23.5°C at Srinagar to 42.6°C at Chitrakoot (Madhya Pradesh), while the minimum temperature ranged from 9.0°C at Srinagar to 28.8°C Sriganganagar and the relative humidity ranged between 30.5% at Sriganganagar to 84.3% at Shillongani (Assam) (Supplementary Table 1). These locations are located in different well-established pulse growing zones, including NWPZ (North Western Plain Zone), NEPZ (North Eastern Plain Zone), CZ (Central Zone), NHZ (North Hill Zone), and SZ (South Zone), which are characterized by different ecological conditions. The individual zones comprised a number of states such as NEPZ, including Eastern Uttar Pradesh, Jharkhand, West Bengal, and Assam; CZ includes Madhya Pradesh, Maharashtra, Chhattisgarh, Bundelkhand region of Uttar Pradesh, Southern Rajasthan and Gujarat; NWPZ consists of Punjab, Haryana, Rajasthan, Delhi, Western Uttar Pradesh, and Plains of Uttarakhand); NHZ comprises Jammu and Kashmir, Himachal Pradesh, Hills of Uttarakhand, and NEH region (Manipur and Tripura); SZ includes Tamilnadu, Karnataka, Orissa, Kerala, Andhra Pradesh, and Telangana states. In this study, there were 4, 6, 7, 11, and 11 locations in NHZ, NEPZ, NWPZ, CZ, and SZ, respectively (see Supplementary Table 1).

### Multi-location testing of genotypes

All the contributing centers (see Supplementary Table 1) conducted preliminary yield trials (PYTs) with local checks during the rainy season of 2015. In the rainy season of 2016, selected genotypes were further tested in large plots in station trials based on the performance of genotypes in PYTs. Likewise, based on genotypes' performance in station trials, one or two promising genotypes were selected for the national testing program (multi-location testing across the states and different growing ecologies). The promising genotypes were submitted for multi-location testing during the rainy season of 2017 throughout the country, under the auspices of the AICRP on MULLaRP (Mungbean, Urdbean, Lentil, Lathyrus, Rajmash, and Pea), Kanpur, India. A total of 34 recently developed diverse mungbean genotypes, along with well-established zone-specific checks, were evaluated for grain yield and other yield attributes. These 34 genotypes were developed through inter- and intra-specific crosses and mutation breeding, contributed by 27 research centers covering 13 agroecological regions of India. Detailed information about the tested genotypes is presented in Supplementary Table 2. As it has been reported in maize, soybean, wheat, and barley that data from single-year and multi-location trials is sufficient to identify superior/inferior genotypes (Yan and Rajcan, 2003), the given materials were tested in a single year at 39 locations.

### Experimental design and general crop management

The preliminary yield trials (PYTs) with local checks were conducted in a randomized complete block design (RCBD) with three replications during the rainy season of 2015. The crop was raised with recommended crop geometry, i.e., a row length of 4 m with a row-to-row distance of 25–30 cm and plant-to-plant spacing of 10 cm. In station trials, the promising genotypes were evaluated in RCBD in three replications during the rainy season of 2016. The individual genotypes were planted in ten rows of 4 m in length. In multi-location trials, the tested genotypes were planted in RCBD with three replications at each location. Each replication had six rows of 4 m in length. The same crop geometry adopted in PYTs was followed in the station and multi-location trials. The experimental field selected at testing sites had flat topography with uniform but representative of fertility for adjoining growing areas. The field was prepared by plowing, harrowing, and planking. In general, the crop was supplied with 20 kg N, 40 kg P_2_O_5_, and 20 kg K_2_O at the time of field preparation. One/two hand weedings were performed to maintain a weed-free crop. Since the crop was raised during the rainy season, life-saving irrigation (1–3 Nos.) was given depending on the rainfall pattern. Necessary plant protection measures were taken to raise a healthy crop. All the selected fields were at considerable distance from population-dense areas and tall buildings.

### Phenological observations

The phenological observations, such as days to 50% flowering (DTF) and days to maturity (DTM), were recorded in each genotype. Days to 50% flowering were assessed in each replication by counting the number of days after sowing until 50% of plants in a plot had one open flower. The DTM was recorded in each replication when pods turned from yellowish brown to black. The cropping period from DTF to DTM was denoted as the reproductive period (RP).

### Estimation of grain yield and 100-seed weight

Before recording the grain yield and 100-seed weight, the harvested seeds were dried under sunlight to achieve 10–12% moisture. Grain yield was recorded in grams per plot in individual replications and further converted into kg ha^−1^. The border rows were not considered in plot yield estimation to remove the border effect. One hundred seeds in three replications were manually counted and weighed to estimate the 100-seed weight.

### Statistical analysis

The significant difference in all treatments was explained through a combined analysis of variance (ANOVA). The grain yield data were subjected to a combined analysis of variance to investigate the effect of genotypes (G), environments (E), and genotype × environment (G × E) interactions. The contributions of G and E, and their interactions to grain yield, were estimated by ANOVA using R-program 3.2.1 (R Development Core Team, Vienna). Heritability in the broad sense (H) was calculated as follows:


$$\begin{array}{l} H=\sigma_{g}^{2}/\sigma_{p}^{2}=1-{\left(SE/SD\right)}^{2}/n, \end{array}$$

where $\sigma_{\text{g}}^{2}$ is the genotypic variance; σ^2^_p_ is phenotypic variance; SE is standard error; SD is standard deviation of genotype means, and n is the number of replicates. The stability of genotypes across the locations was approximated numerically and graphically using HA-GGE biplot analysis (Yan and Holland, 2010), which displays the graphical analysis of multi-environment data through “genotype effect” (G) and the “genotype × environment” (G × E) effect. The HA-GGE biplot analysis was performed by the R-program 3.2.1 using calculated heritability.

The basic model for the GGE biplot (Yan and Hant, 2002) is given below:


$$\begin{array}{l} Y_{ij}-\mu -\beta_{j}=\;\overset{K}{\underset{k=1}{\sum }}\lambda_{k}\xi_{ik}\eta_{jk}+\varepsilon_{ij}, \end{array}$$

where Y_ij_ is the measured mean of genotype i in environment j, μ is the grand mean, β_j_ is the main effect of environment j, with μ + β_j_ being the mean grain yield across all genotypes in environment j, λ_*k*_ = the singular value (SV) of k^th^ principal component (PC), ξ_ik_ are eigenvectors of genotype i for PC_k_, η_jk_ are eigenvectors of environment j for PC_k_, ε_ij_ is the residual associated with genotype i in environment j, and K is the number of PC axes retained in the model [K ≤ min (g, e) and K = 2 for a 2-dimensional biplot].

The GGE biplots were developed by plotting the first two symmetrically scaled principal components (PC1 and PC2, also referred to as primary and secondary effects, respectively) derived from the different environment-centered data (Yan and Hant, 2002). When the data was scaled with factor s_j_ (referred to as data scaling), the general formula for the GGE biplot was restructured as follows:


$$(Y_{ij}-\mu -\beta_{j})/s_{j}=\;\sum_{l=1}^{k}g_{il}e_{lj}\;+\;\varepsilon_{ij}$$

In the heritability adjusted GGE-biplot, the data are scaled by the factor $\frac{s_{j}}{\sqrt{H_{j}}}$, where *s*_*j*_ is the standard deviation of the jth environment, and *H*_*j*_ is the heritability in the broad sense in environment j.

The discrimination authority of an environment in the HA-GGE biplot is approximately equivalent to the vector length of the respective environment, which is the line connecting the origin and the test environment points, and representativeness is approximately equal to the cosine of the angle between the environment vector and the average environment vector (Yan and Holland, 2010; Badu-Apraku et al., 2012). The desirability index of an environment is approximately equal to the negative projection of the environment vector onto the average environment vector axis. The interrelationship between the test locations was measured through the cosine of angles between the vectors of test environments (a test environment and a target environment) (Yan and Holland, 2010; Luo et al., 2015a). Relatedness between the genotypes and environments was calculated using the Ward method and represented through a hierarchical cluster. The HA-GGE biplot analysis was done using the R software (R Development Core Team, Vienna).

## Results

### Weather variables in testing locations

The study sites (*n* = 39) markedly differ in weather variables (temperature, relative humidity, and rainfall) during the cropping season (Supplementary Table 1). The altitude of the locations ranged between 12.0 m (Mohanpur and Navsari) and 1,617.0 m (Srinagar). The rainfall received during the crop season varied between 5.25 mm (Sriganganagar) and 1,593 mm (Jagdalpur). The highest Tmax was recorded in Chitrakoot (42.6°C), while Srinagar recorded the least Tmax of 23.5°C, with a mean Tmax of 32.5°C. Similarly, the Tmin ranged between 9.0°C (Srinagar) to 28.8°C (Sriganganagar), with a mean of 23.4°C. The RH values showed a marked variation within the locations, with the highest in Shillongani (84.0%) and the lowest in Sriganganagar (30.5%), with an average of 66.6%.

### Site-specific changes in crop phenology

**Figure 2:**
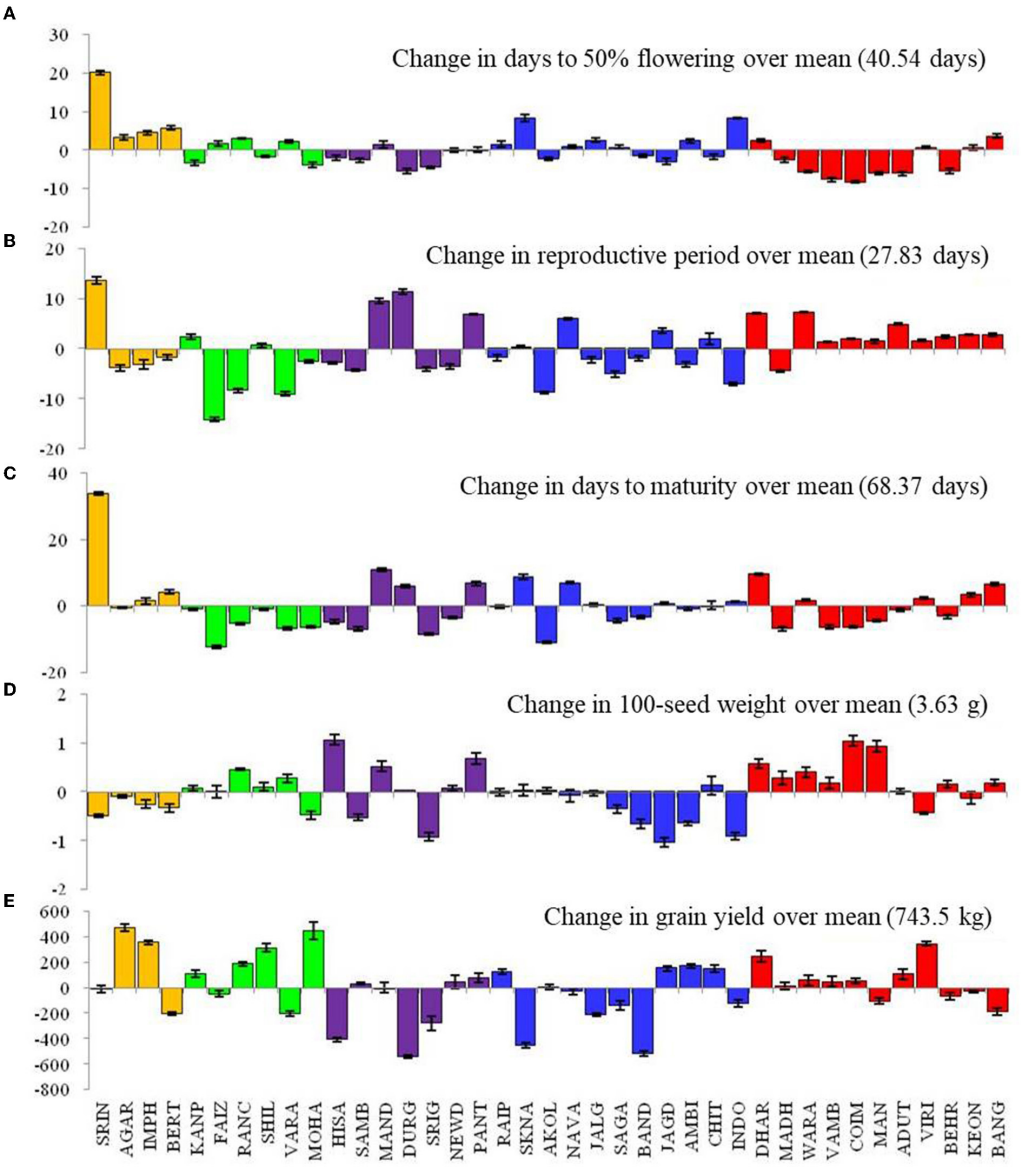
Changes in crop phonological events **(A–C)**, 100-seed weight **(D)**, and grain yield **(E)** of mungbean across 39 locations. Please see Supplementary Table 1 for environment (E1–E39) detail.

Results showed the prominent influence of the environment on phenological events such as DTF, DTM, and RP (Figure 2). The DTF was increased by > +20 days in Srinagar, and by ≥5 days in Berthin, SK Nagar, and Indore; it was decreased in Durgapura, Warangal, Vamban, Coimbatore, Mandya, Aduthurai, and Berhampur by 5 days as compared to the overall mean value of 40.5 days. The genotypic variations in DTF, as measured by the coefficient of variation (CV), were prominent (15.1–23.1, mean = 18.44), highest for the genotype LGG 607 and lowest for the genotype VGG 16-055 (Table 1).

**Table 1:** Associations of crop phonology with grain yield according to linear mixed regression in different testing locations.

**Genotype**	**Coefficient of variation (CV)**			***n***	**Regression coefficient**			**Multiple R**	***p*** **value**			
	**GY**	**DTF**	**RP**		**Intercept**	**DTF**	**RP**		**Regression**	**Intercept**	**DTF**	**RP**
AKM 12-24	41.8	21.7	24.0	39	205.8	5.44	11.26	0.27	0.308	0.630	0.531	0.153
AKM 12-28	40.6	21.6	22.9	39	668.4	−1.45	3.98	0.18	0.608	0.068	0.869	0.323
BM 2012-9	47.0	21.3	27.2	39	429.9	4.35	0.18	0.08	0.910	0.370	0.667	0.978
COGG 13-39	44.8	21.1	24.8	39	1047.4	−9.24	0.98	0.20	0.537	0.023	0.277	0.901
DGG 7	53.4	20.7	22.5	39	541.7	−0.27	2.89	0.14	0.734	0.143	0.975	0.435
IGKM 2016-1	46.3	21.1	22.9	39	1295.9	−10.86	−7.01	0.28	0.274	0.003	0.158	0.402
IPM 410-9	38.6	21.4	30.3	39	1049.3	−2.21	−6.21	0.17	0.641	0.035	0.840	0.356
IPM 512-1	36.3	20.9	24.2	39	1402.8	−11.98	−4.59	0.28	0.273	0.001	0.148	0.540
JAUM 0936	33.9	19.3	22.5	39	484.9	0.87	9.96	0.22	0.452	0.302	0.931	0.212
JLM 302-46	42.9	19.6	25.2	39	624.8	−0.68	0.93	0.03	0.985	0.133	0.934	0.892
KM 2355	36.7	19.3	22.1	39	536.9	0.28	7.28	0.17	0.625	0.162	0.970	0.336
LGG 607	41.3	23.1	40.5	39	713.2	3.53	−4.12	0.14	0.738	0.065	0.647	0.564
MDGGV-18	35.0	18.9	22.5	39	1184.3	−14.48	5.50	0.33	0.174	0.009	0.100	0.443
MGG-387	39.3	18.8	25.0	39	954.8	−2.35	−1.50	0.06	0.941	0.020	0.760	0.844
MH 1323	33.2	18.7	22.2	39	709.0	0.53	1.45	0.03	0.985	0.153	0.954	0.866
ML 2479	39.4	17.7	22.4	39	1037.9	−8.74	2.77	0.17	0.645	0.031	0.384	0.735
NDMK 16-324	37.6	18.0	22.9	39	945.3	−11.94	10.82	0.32	0.198	0.047	0.213	0.204
NMK 15-08	39.2	17.8	22.9	39	413.3	1.90	8.51	0.16	0.686	0.414	0.857	0.404
NVL-855	46.7	18.3	22.5	39	930.3	−1.16	−5.83	0.12	0.801	0.043	0.900	0.517
OBGG 56	42.3	17.8	22.1	39	869.4	−9.47	10.55	0.28	0.270	0.048	0.314	0.173
OBGG 58	38.2	18.4	25.6	39	575.7	−1.85	8.30	0.18	0.584	0.209	0.844	0.310
PM 14-11	41.4	17.4	23.1	39	726.4	−11.27	15.69	0.47	0.022	0.053	0.098	0.038
PM 14-3	38.3	16.5	21.0	39	914.9	−2.33	−2.22	0.07	0.933	0.035	0.764	0.793
Pusa M 1771	37.3	17.0	23.0	39	1023.3	−8.53	2.80	0.24	0.412	0.010	0.234	0.702
Pusa M 1772	33.9	16.5	22.6	39	720.0	1.59	−1.80	0.06	0.949	0.095	0.846	0.820
RMB 12-07	39.2	16.6	23.5	39	816.5	−1.22	−1.71	0.04	0.971	0.064	0.879	0.833
RMG 1097	36.4	16.1	22.0	39	442.3	2.79	5.45	0.12	0.786	0.308	0.749	0.533
SKNM 1502	39.9	16.3	21.2	39	852.3	−0.27	−4.58	0.13	0.758	0.020	0.971	0.460
SKNM 1504	31.5	16.2	23.3	39	836.0	−3.59	2.31	0.08	0.909	0.088	0.724	0.799
SML 1808	39.4	15.6	21.3	39	518.7	3.58	2.98	0.10	0.847	0.196	0.655	0.699
SVM-6133	37.3	16.1	23.4	39	717.8	−1.50	2.06	0.04	0.976	0.202	0.899	0.853
TMB 126	**52.5**	16.2	22.7	39	937.9	−4.79	−2.42	0.20	0.516	0.001	0.385	0.747
VGG 16-036	44.0	15.7	28.5	39	977.4	−7.07	4.21	0.16	0.655	0.036	0.435	0.642
VGG 16-055	36.5	15.1	20.9	39	373.9	3.61	5.50	0.18	0.607	0.348	0.674	0.361

The locations Srinagar, Mandya, SK Nagar, and Dharwad, took extended crop duration (≥+8 days) over the average value of 68.4 days. On the contrary, the locations of Faizabad, Sriganganagar, and Akola had shortened (>8 days) crop duration over the overall average DTM. Prolong RP has been observed at the locations Srinagar (+13 days), Mandya, Durgapura, Pantnagar, Navasari, Dharwad, and Warangal (>5 days). Likewise, the locations of Faizabad, Ranchi, Varanasi, Akola, Sagar, and Indore had shorter RP (>5 days) than the average RP value of 27.8 days (Figure 2). The coefficient of variation for RP in all genotypes ranged from 20.9 (VGG 16-055) to 40.5 (LGG 607), with a mean value of 23.93. The genotypes IPM 410-9, VGG 16-0.36, and BM 2012-9 exhibited higher CV (>27%). The genotypic variation for grain yield measured by CV oscillated from 31.5 (SKNM 1504) to 53.4 (DGG 7) (Table 1).

**Figure 3:**
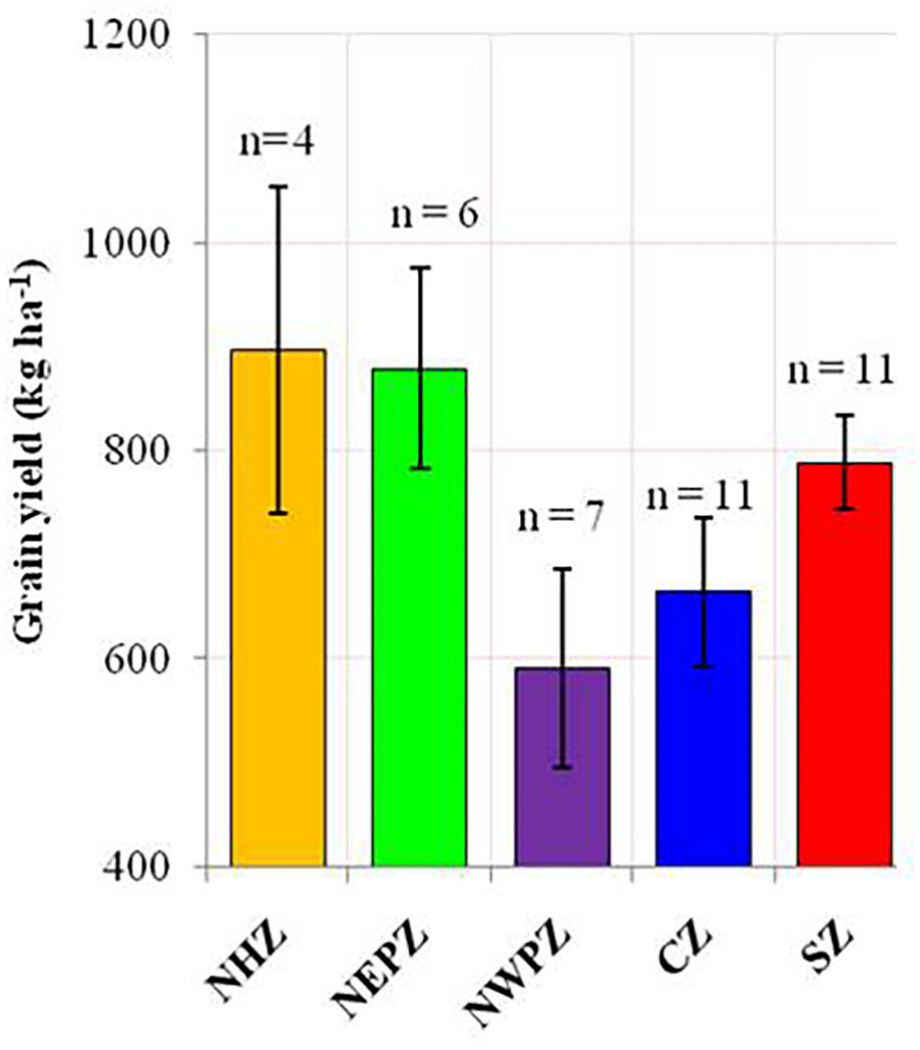
The average productivity of mungbean in different agroecological regions of India. The error bar represents the standard error of means. North Hill Zone (NHZ), North Eastern Plain Zone (NEPZ); North Western Plain Zone (NWPZ); Central Zone (CZ); South Zone (SZ).

The ANOVA results demonstrated that the total variability in grain yield parameter was majorly contributed by the environment (54.17%), followed by the interactive effect of genotype × environment (29.65%) and genotype (2.98%), and all the effects were significant at *p* < 0.001 (Table 2). The linear mixed model was developed by considering the grain yield as a dependent variable and DTF and RP as independent variables. The results revealed that environment-induced variations in DTF and RP had a non-significant influence on the yield of mungbean genotypes except for the cultivar PM 14-11 (Table 1). Particular to the genotype PM 14-11, grain yield was influenced by changes in phenological events, being higher for the parameter RP over DTF. Likewise, the mean yield data across the genotypes also illustrated that the effects of RP and DTF had no direct influence on the grain yield of the tested mungbean genotypes. The highest average grain yield was achieved in NHZ (897 kg ha^−1^), followed by NEPZ (879.63 kg ha^−1^), SZ (789.31 kg ha^−1^), whereas the lowest was observed in NWPZ (591.29 kg ha^−1^) (Figure 3).

**Table 2:** Analysis of variance for grain yield (kg ha^−1^) in 34 genotypes of mungbean evaluated over 39 environments in India.

**Source of variation**	**Degrees of freedom**	**Sum of squares**	**Mean sum of squares**	**Contribution (%)**	**F-value**	***p* value**
Replication	2	94,411.40	47,205.70	0.02	2.21	*ns*
Environment (E)	38	232,686,549.90	6,123,330.26	54.17	286.78	<0.01
Genotype (G)	33	12,803,521.30	387,985.49	2.98	18.17	<0.01
G × E interaction	1254	127,348,599.90	101,553.91	29.65	4.76	<0.01
Error	2650	56,582,602.50	21,351.93	13.17		
Total	3977	429,515,685.10				

### Inter-relationship between crop traits and weather parameters

**Figure 4:**
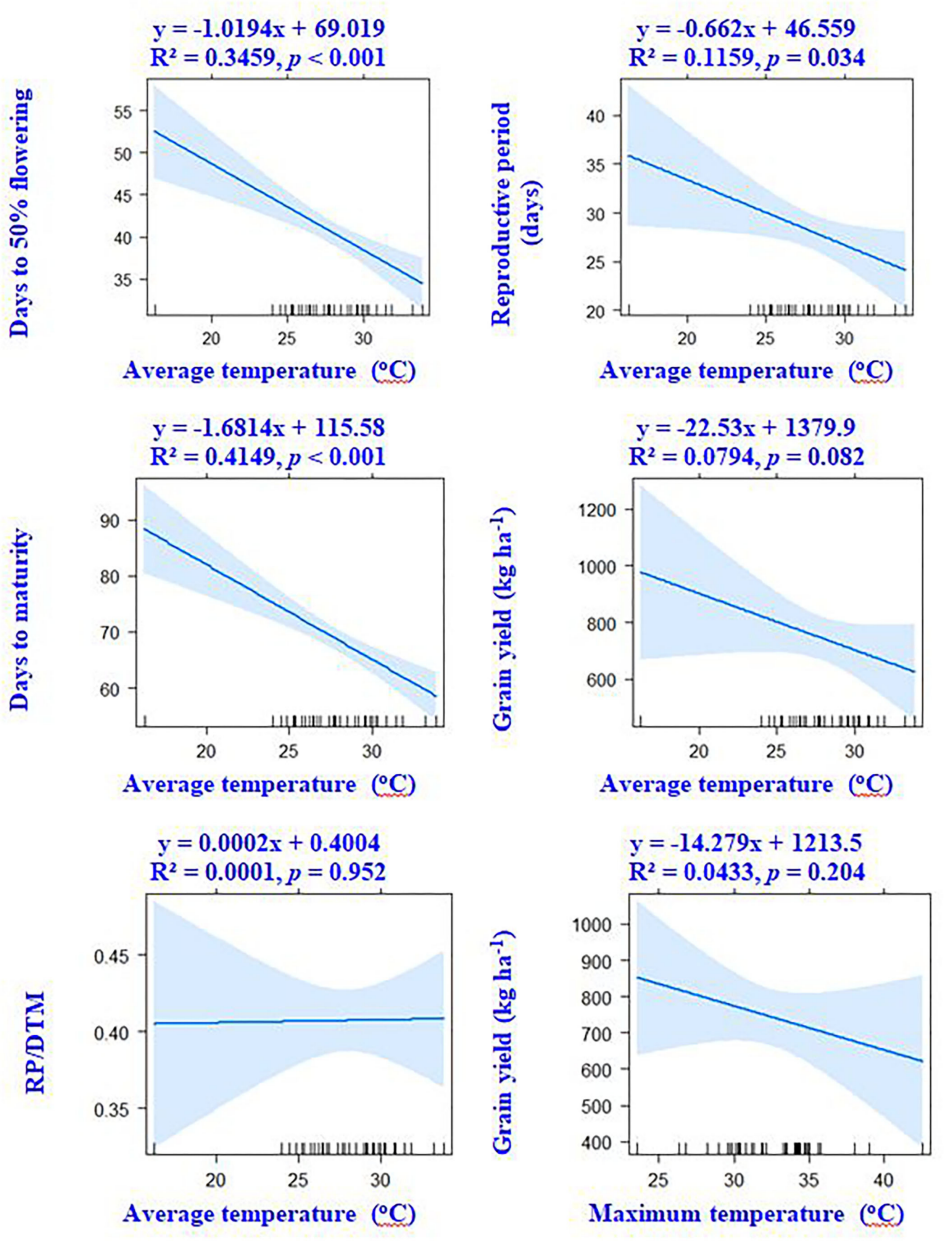
Associations between crop phenology, temperature variables, and grain yield of mungbean according to linear regression models.

**Figure 5:**
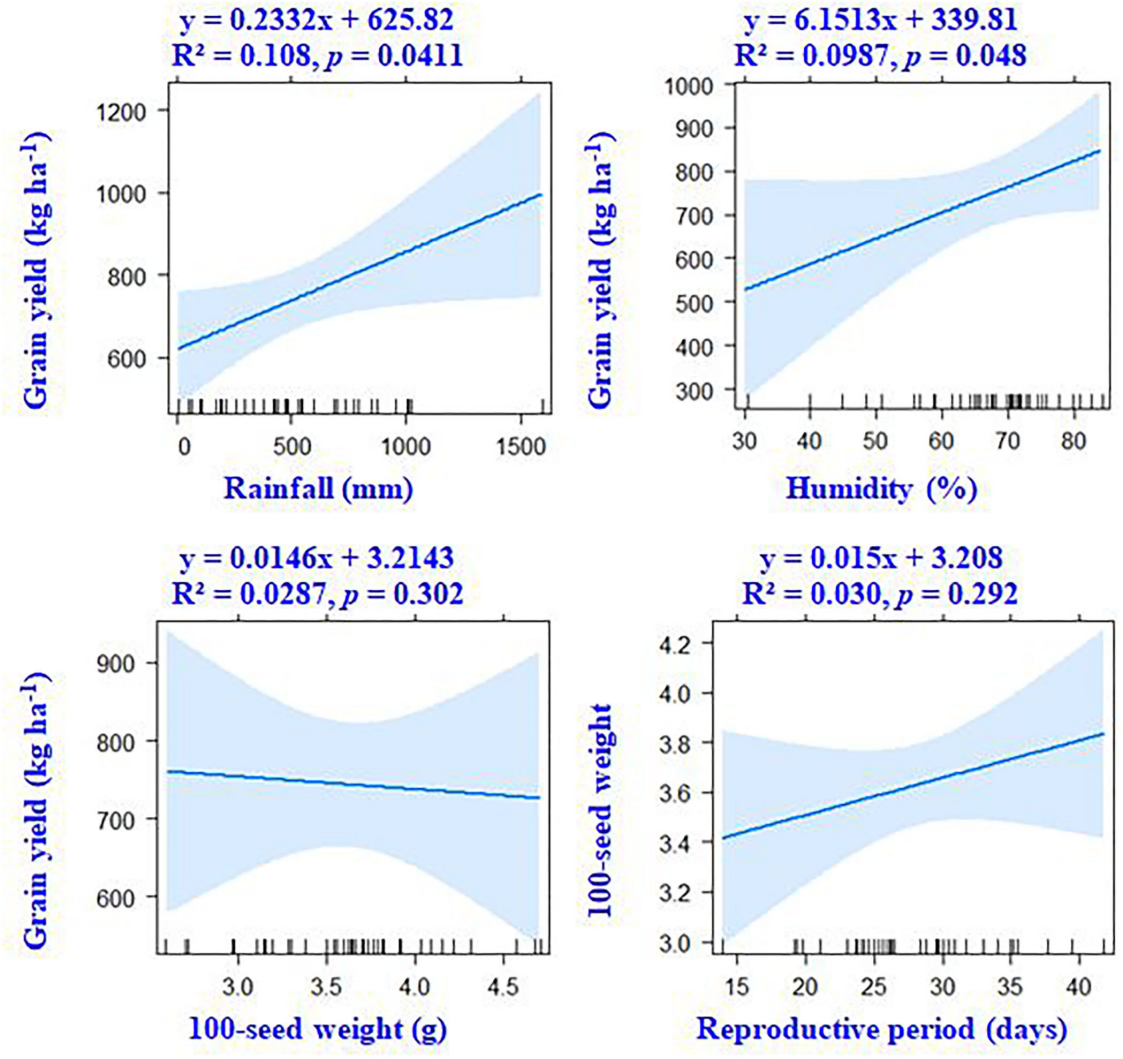
According to linear regression models, there are associations between grain yield, weather parameters, and the seed weight of mungbean.

Correlation results showed that DTF, RP, and DTM had a negative association with ambient temperature. The average and maximum temperature had a non-significant (*p* > 0.05) correlation with grain yield (Figure 4). However, significant positive associations of grain yield were observed with crop season rainfall (*r* = +0.33, *p* < 0.05) and relative humidity (*r* = +0.31, *p* < 0.05). Results revealed that the environmental-induced changes in 100-seed weight and RP had non-significant correlations with grain yield (*p* > 0.05) (Figure 5).

### Multivariate analysis (PCA, HA-GGE biplot, cluster analysis)

**Figure 6:**
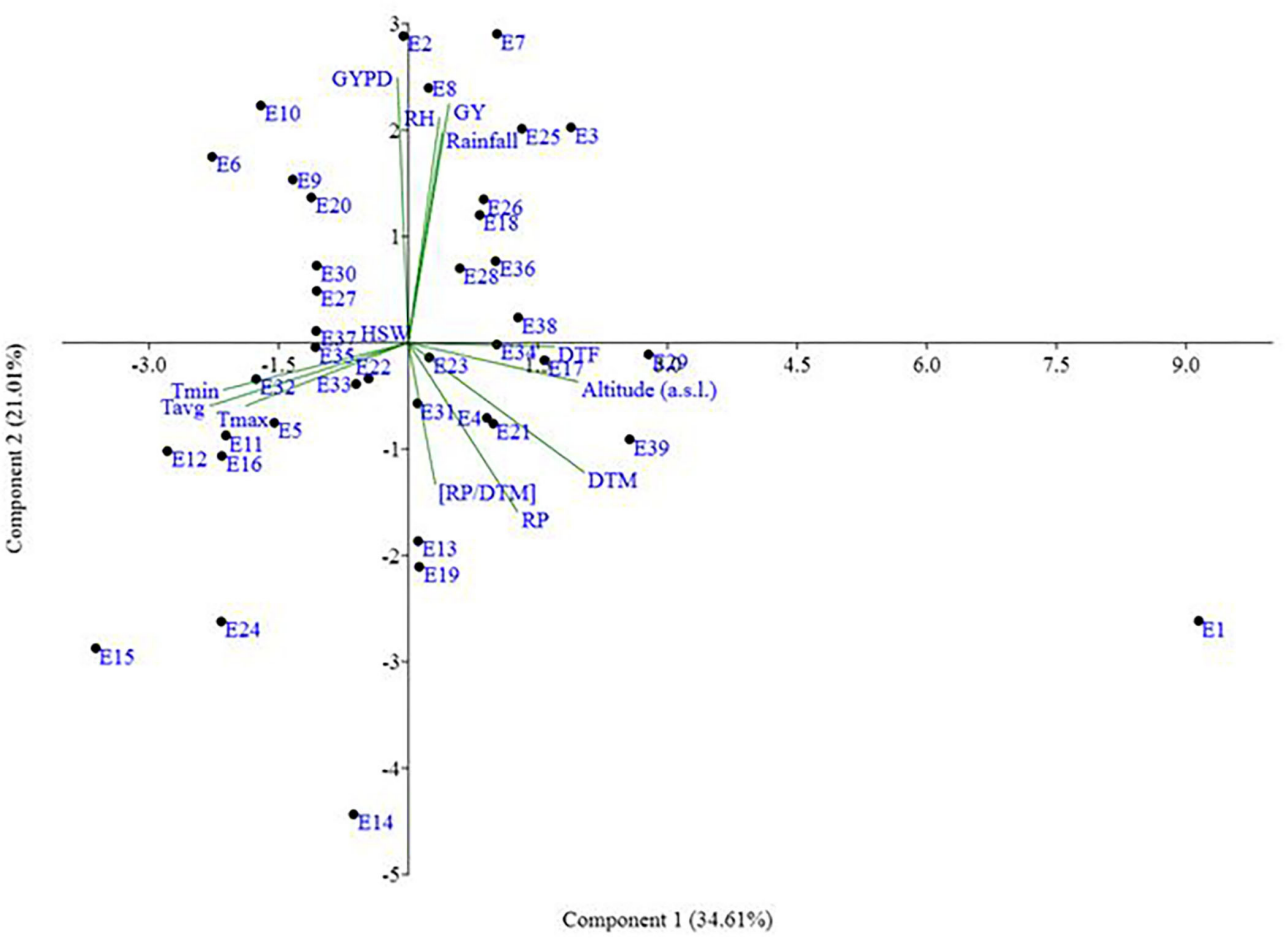
Scatter plot environments on PCA coordinates and biplot presentation of crop phenology, seed weight, grain yield parameters, and weather variables. Please see Supplementary Table 1 for environment (E1–E39) detail.

**Figure 7:**
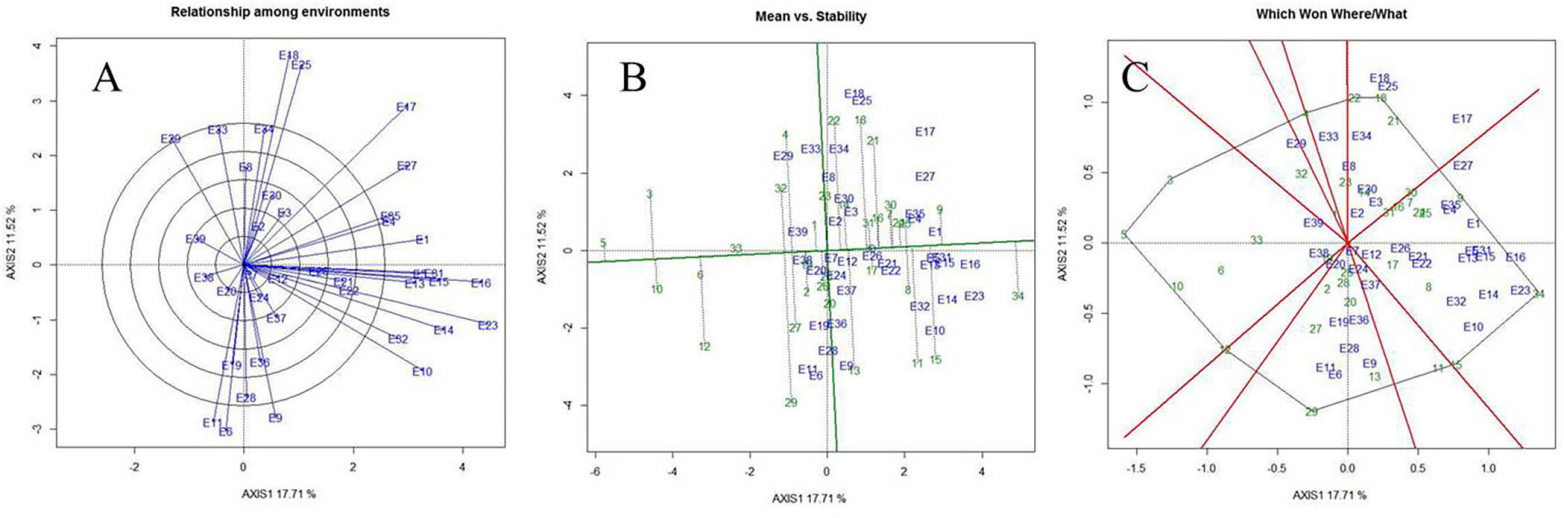
Relationship among the test environments **(A)**, mean vs. stability **(B)**, and 'Which-won-where' **(C)** view of test locations based on heritability-adjusted GGE (HA-GGE) biplot analysis of 34 mungbean genotypes across 39 testing locations. No transformation of data (transform = 0); and data were centered by means of the environments (centering = 2). The biplot was based on “Column metrics preserving', i.e., genotype and environment-focused singular-value partitioning. Therefore, it is most appropriate for illustrating the relationship between genotypes and environments. Numbers correspond to environment and genotypes, as listed in Supplementary Tables 1, 2.

**Figure 8:**
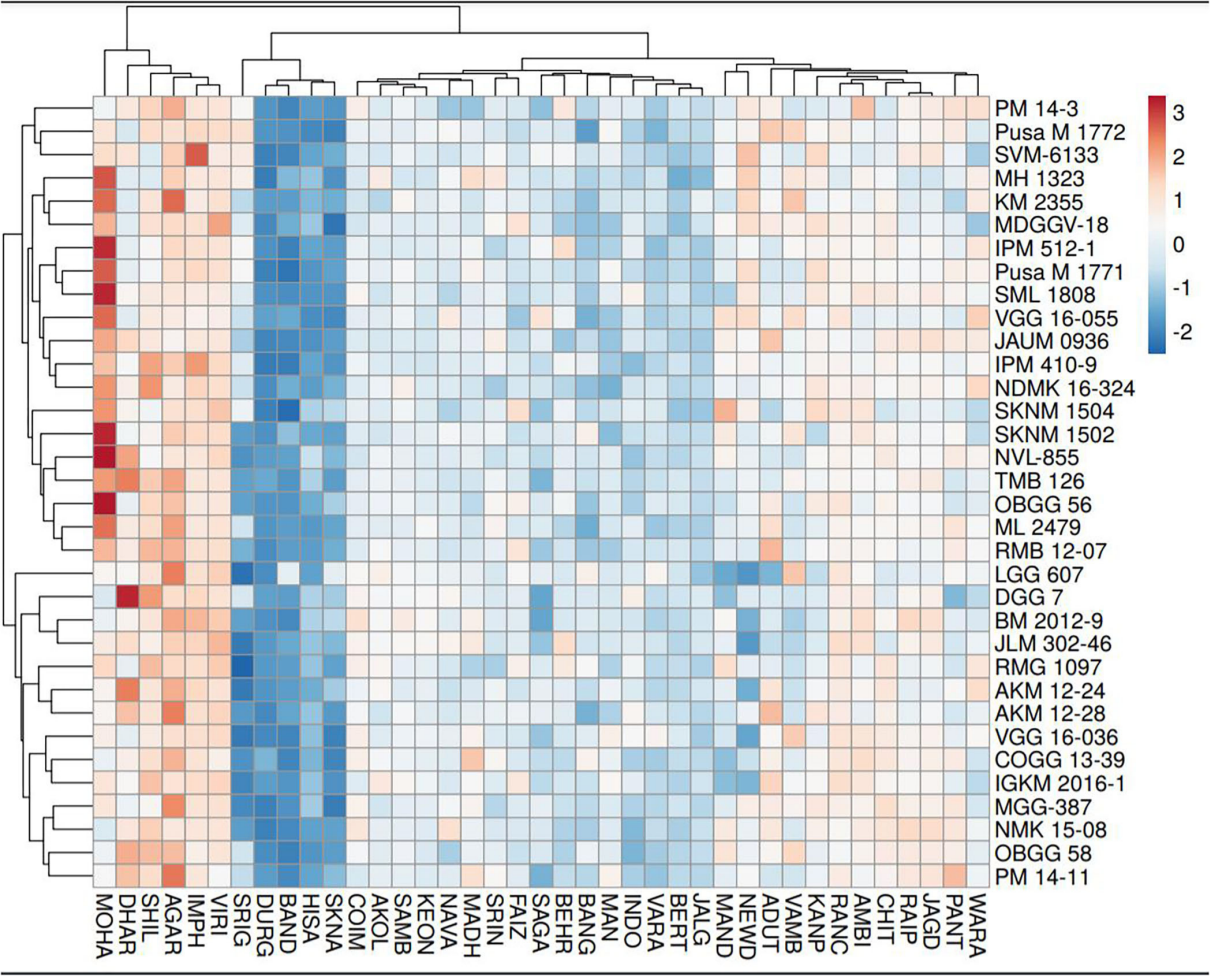
Hierarchical cluster analysis explaining the relationship between mungbean genotypes (*n* = 34) for grain yield across different testing locations (*n* = 39).

As per the PCA result, the crop season rainfall and grain yield had a strong positive association (a lower acute angle between the vector axis represents a higher scale of association), while both these variables varied largely among the environments. The altitude effect was marginal on the grain yield of mungbean across Indian climates. Minimum and maximum temperatures had a negative association with DTF, while location altitude and DTF exhibited positive associations. Similarly, grain yield exhibited negative associations with RP as well as a [RP/DTM] ratio (Figure 6). The vector axis length of the PCA graph revealed that environments like Faizabad (E6), Mohanpur (E10), Varanasi (E9), Ranchi (E7), Shillongani (E8), Agartala (E2), Imphal (E3), and Jagdalpur (E25) lead to increased variability within the genotypes for grain yield. The HA-GGE biplot results (Figure 7) showed that the influence of environments on mungbean yield was much more diverse and variable across the studied environments. The mega environments/locations group [Faizabad (E6), Varanasi (E9), Hisar (E11), SK Nagar (E19), Virinjipuram (E36), Indore (E28)], [Mohanpur (E10), Mandore (E13), Durgapura (E14), Sriganganagar (E15), New Delhi (E16), Sagar (E23), Warangal (E31), Vamban (E32), Navasari (E21), Jalgaon (E22), Sagar (E23)] and [Shillongani (E8), Madhira (E30), Mandya (E34), Agartala (E2), Imphal (E3)] had more or less equivalent scale of impact on mungbean yield potential. According to the results, the tested genotypes exhibited wider variability regarding mean and stability of yield across locations, where genotype VGG 16-055 (contributed by Vamban, SZ) was the most productive, while genotype DGG 7 (contributed by Dharwad, SZ) was the least productive among the genotypes. Genotypes VGG 16-036 (33), DGG 7 (5), AKM 12-24 (1), NDMK 16-324 (17), IGKM 2016-1 (6) were highly stable across locations, while genotypes COGG 13-39 (4), PM 14-11 (22), SKNM 1504 (29), MDGGV-18 (13), KM 2355 (11), and OBGG 58 (21) exhibited a greater scale of yield instability across the locations. Considering mean vs. stability, VGG 16-055 (34) and JAUM 0936 (9) were identified as the ideal genotypes among the tested genotypes. The representativeness scale within the studied environments ranges from −0.99 to +1.0, and the discriminative power from 0.56 to 4.59. The desirability index, which represents the overall performance of a location based on its “discriminatory” power and “representativeness,” varied between −1.22 and +4.46. The locations of Sagar (CZ), New Delhi, Sriganganagar, Durgapura (NWPZ), Warangal (SZ), Srinagar (NHZ), Kanpur, and Mohanpur (NEPZ) as the ideal testing environments demonstrated high efficiency in the selection of new genotypes with wider adaptability (Figure 8). The two-way hierarchal clustering of genotype and environment also witnessed an ample amount of variability available in genotype and environment performance in terms of grain yield in mungbean (Figure 8).

## Discussion

Results demonstrated that the agro-climatic conditions have a considerable influence on crop expression in terms of phenological events and grain yield. In India, the improved breeding lines bred at different agro regions undergo a national level multi-location trial before being released as a superior cultivar with considerable yield advantage or any particular trait of interest over the local or national check(s). The site-specific performance of a crop is mostly dependent on environmental (temperature, rainfall, humidity, sunshine hours) and edaphic conditions. The results demonstrate that low temperature and high altitude environments such as Srinagar, Imphal, and Berthin had extended vegetative stages (+5 to +20 days), resulting in longer crop duration. The yield potential of low temperature and high altitude locations remains comparable to or even higher than the average of the overall locations. This implies that for rainy season mungbean, the hilly regions could be considered as potential niches, which currently have very low mungbean acreage (Praharaj and Singh, 2019). Low-temperature environment-mediated increase in crop duration may be due to slow growth rate and other physiological functions, which may be described by the thermo-sensitivity of the crop (Pratap et al., 2019). A similar increase in crop duration was reported for the cool-season legumes from elevated temperate agro-regions (Wright et al., 2021). On the other hand, the south Indian locations (Dharwad, Madhira, Warangal, Vamban, and Coimbatore) recorded shorter vegetative periods with prolonged reproductive periods, which may be ideal crop phenology for higher productivity of mungbean. The 100-seed weight was increased in these locations, indicating improved grain filling and source-sink efficiencies likely attributed to the extended reproductive period. According to the results, significant yield reduction was observed in the locations of Durgapura, SK Nagar, Banda, Hisar, Sriganganagar, and Sagar, which belong to the northern, northwestern, and central part of the country. These locations are characterized as arid regions, and therefore, the climatic conditions might be the major limiting factor for sustaining crop yield in these locations. Data revealed that 100-seed weight was the lowest in this region, possibly due to forced maturity-induced shortening of the grain filling period. The soil of arid regions is reported as less fertile (low soil organic carbon <3 g kg^−1^) (Kumar et al., 2021; Moharana et al., 2021), which could have exaggerated the yield penalty. In this study, soil attributes were not considered; however, a major influence of soil properties on crop performance, particularly on the yield is, therefore, expected.

Our results suggest that variable climatic conditions could influence crop phenological events such as flowering time, grain filling period, and crop maturity duration. For instance, the higher ambient temperature in NWPZ advanced the flowering time, indicating increased thermal sensitivity of the mungbean crop. The PCA results also confirm the negative association between the ambient temperature and flowering time. The negative association between the reproductive period and ambient temperature suggests either forced maturity under high-temperature environments or an extended grain-filling period in relatively cooler regions, such as the northern hill and southern zones. Non-synchronous maturity is a common phenomenon in rainy season legumes like mungbean, urdbean (*Vigna mungo* L.), and cowpea (*Vigna unguiculata* L.) owing to indeterminate growth habit, particularly if the moisture conditions are favorable at the later growth stages (Ha et al., 2020). Often, favorable conditions during the reproductive period result in the second flush of flowers, their subsequent fertilization, and seed/pod set, which extends the maturity duration (Marwiyah et al., 2021). Seasonal rainfall and humidity have shown positive associations with grain yield, suggesting that the higher or optimum rainfall areas are more favorable for mungbean, leading to higher yields. The PCA result confirms the influence of rainfall and relative humidity on the grain yield. Our results suggest that the changes in crop phenological events such as flowering time, reproductive period, and full crop season duration have no specific influence on the grain yield potential of mungbean in Indian climates during the rainy season. Although, the changes in the vegetative and grain filling period have a notable influence on crop productivity of winter crops like field pea, chickpea, and lentils (Parihar et al., 2022). There was a notable difference in 100-seed weight across the environment; however, no association of 100-seed weight with grain yield suggests seed weight may not necessarily be a yield determinant.

**Figure 9:**
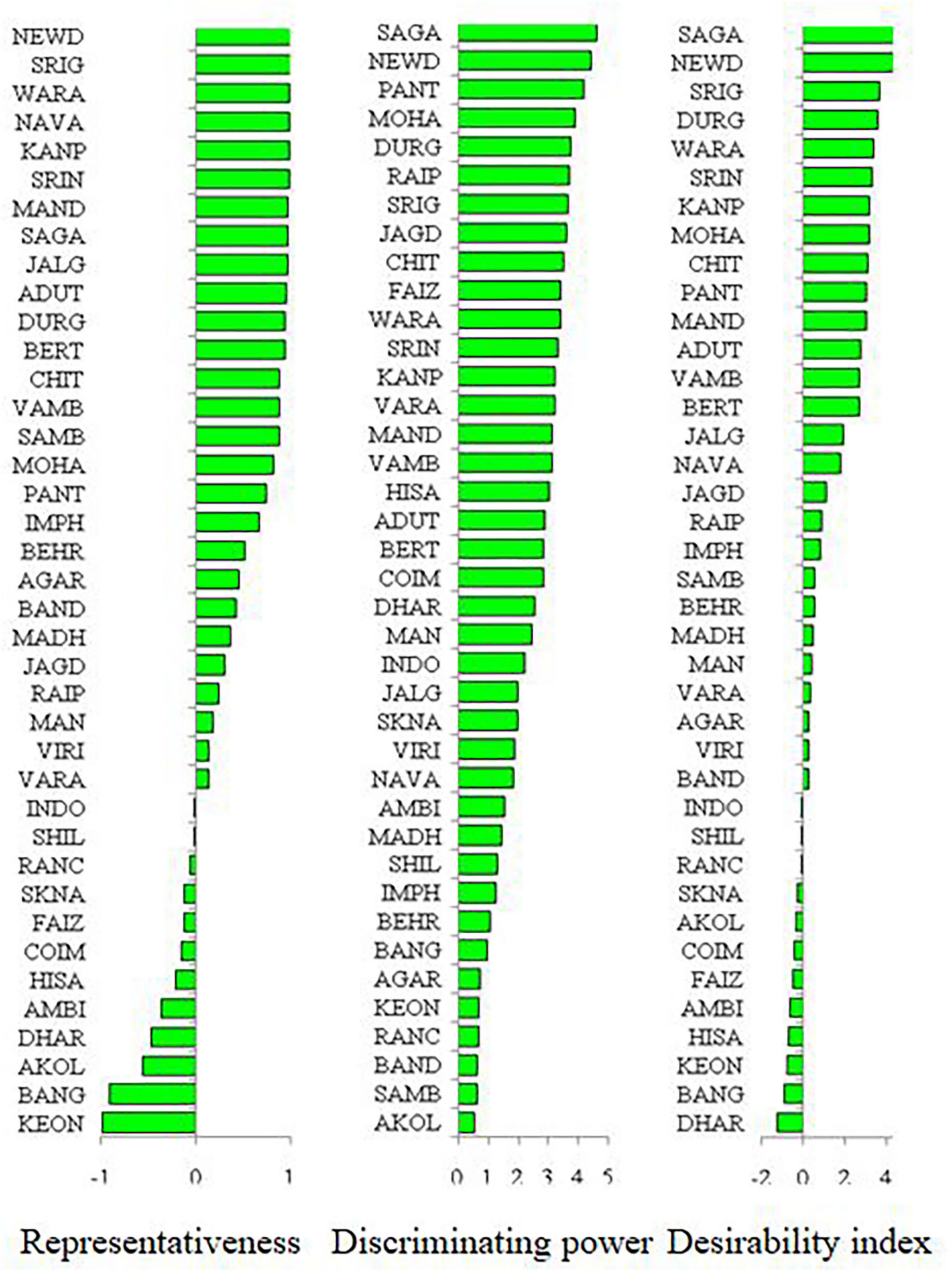
Representativeness, discriminating power, and desirability index of different locations. Please see Supplementary Table 1 for environment (E1–E39) detail.

Mungbean productivity in India is very unstable and quite low (12 out of 39 locations had <700) as compared to other countries (Anonymous, 2019). Consequently, the selection of high-yielding, stable genotypes and their proper use in a breeding program is the most practical approach to increase mungbean productivity. Therefore, an in-depth understanding of the extent and reasons for GEI is extremely valuable for designing breeding objectives, identifying ideal test locations, and making varietal recommendations (Yan and Hunt, 2001). It is also essential for approximating the adaptability and stability of the genotypes. Based on our results, the G × E interaction is responsible for the highest magnitude of variability, indicating a greater site-specific response of genotypes. Considering this, a cultivar bred for a particular region may not necessarily perform well across the country level. Therefore, it is suggested to increase the number of locations in appraisal trials to cover all available diverse ecological conditions. In addition, the identified suitable genotypes for a particular location should be planted in that location to exploit positive GEI effects. Hence, efforts must be directed toward identifying potential locations (mega environments) for a breeding program and selecting strategic testing locations for cultivar evaluations. In the study, the HA-GGE biplot was applied to assess 39 locations considering grain yield. The locations of Sagar (E23), New Delhi (E16), Sriganganagar (E15), and Durgapura (E14) were highly representative and discriminative of their target environment (Figure 9). These ideal locations demonstrated high efficiency in selecting high-performing genotypes with wider adaptability based on high discrimination power and representativeness (Yan et al., 2011; Asfaw et al., 2012; Ullah et al., 2012; Luo et al., 2015b). Instead, locations with high discriminating power but low representativeness can be used in the culling of unstable genotypes and should be given utmost importance for yield testing in the mungbean. Similarly, the discriminating versus representativeness view of the GGE biplot has been used to assess the testing environments for mungbean (Yan et al., 2011; Asfaw et al., 2012; Ullah et al., 2012; Alam et al., 2014; Baraki et al., 2020). The “desirability index” of a testing location is the cumulative demonstration of a particular location's performance based on “discriminatory” power and “representativeness” (Yan et al., 2011; Parihar et al., 2018; Singh et al., 2020). The desirability index suggested New Delhi (E16) as a useful test environment, followed by Sagar (E23) and Sriganganagar (E15) regarding selection response (Fig 9). In contrast, the least desirable environments for mungbean grain yield were Dharwad (E29) and Banglore (E39). It could be helpful to judge the comparative efficiency of the test environment for indirect selection of a well-known target environment (Yan, 2010; Yan and Holland, 2010). Additionally, the desirability index of a genotype can be used as an imperative criterion for dropping redundant test environments. Finally, considering the three parameters, i.e., discriminativeness, representativeness, and desirability index, New Delhi (E16) and Sagar (E23) were ideal environments for testing of mungbean genotypes along with Sriganganagar (E15), Durgapura (E14), and Srinagar (E1).

## Conclusions

The study concluded that the performance or productivity potential of the mungbean genotypes differed substantially across the different regions of India. The results demonstrated that the changes in crop phenology, such as days to flowering and maturity duration with locations, had no direct influence on mungbean productivity, while the weather variables (rainfall, relative humidity, and temperature) are the yield determinants for the rainy season mungbean. Further, the study suggested horizontal expansion of mungbean could be possible in the northern hill zone where the acreage of mungbean is presently very low. The south zone showed contrasting results where the extended grain-filling period improved seed development (high seed weight) and yield sustainability. Considering mean vs. stability, VGG 16-055 and JAUM 0936 were identified as the ideal genotypes among the tested genotypes. Among the tested locations, New Delhi, Sagar, Sriganganagar, Durgapura, Srinagar, Kanpur, and Mohanpur were identified as the ideal location(s) for the selection of superior genotypes with wider adaptability across India.

## Data availability statement

The raw data supporting the conclusions of this article will be made available by the authors, without undue reservation.

## Author contributions

AKP and SG conceptualized and designed multi-location trials, coordinated the trials, and wrote the original draft of the manuscript. KKH performed the data interpretation and did the manuscript writing, as well as editing. AL performed the data interpretation and manuscript editing. DSG contributed to manuscript editing. DSi and RKu contributed the statistical analysis. AKS contributed the data compilation. RV, MSJ, SPD, JD, RKY, BSJ, BRC, OPK, VP, HKD, RKP, MK, PK, CSM, HKB, MNS, AD, ANP, HCN, VK, SDR, DAC, MHP, RRK, JK, SPM, HK, IS, SM, DK, NM, MBG, MP, PJR, DSh, AMP, PM, KI, and SD conducted trials and performed phenotyping. All authors contributed to the article and approved the submitted version.

## Conflict of interest

The authors declare that the research was conducted in the absence of any commercial or financial relationships that could be construed as a potential conflict of interest.

## Publisher's note

All claims expressed in this article are solely those of the authors and do not necessarily represent those of their affiliated organizations, or those of the publisher, the editors and the reviewers. Any product that may be evaluated in this article, or claim that may be made by its manufacturer, is not guaranteed or endorsed by the publisher.
