# Supplementary material for: Multi-location evaluation of mungbean (Vigna radiata L.) in Indian climates: Ecophenological dynamics, yield relation, and characterization of locations
Source: Front Plant Sci. 2022 Sep 20;13:984912. doi: 10.3389/fpls.2022.984912 (PMC9530336; doi:10.3389/fpls.2022.984912)
Supplement: Supplementary file 1 [file Data_Sheet_1.doc]

**Supplementary Table 1ǀ**Climate variables of the 39 test locations during crop duration in *kharif*/rainy season of 2017

| Zone | Location/  environment | Biplot code | Latitude (°N) | Longitude  (°E) | Altitude (a.s.l.) | Rainfall | Tmax | Tmin | RH | AER | Location Abbreviation |
| --- | --- | --- | --- | --- | --- | --- | --- | --- | --- | --- | --- |
| North Hill Zone  (NHZ) | Srinagar | E1 | 33.98 | 74.80 | 1617.0 | 53.75 | 23.50 | 9.00 | 48.50 | WHR | SRIN |
|  | Agartala | E2 | 25.53 | 91.55 | 16.2 | 682.97 | 31.17 | 24.17 | 80.83 | EHR | AGAR |
|  | Imphal | E3 | 24.49 | 93.57 | 810.0 | 1006.00 | 29.60 | 21.10 | 61.45 | EHR | IMPH |
|  | Berthin | E4 | 31.24 | 76.38 | 644.0 | 952.80 | 33.39 | 22.04 | 55.75 | WHR | BERT |
| North Eastern Plain Zone  (NEPZ) | Kanpur | E5 | 26.44 | 80.33 | 133.0 | 197.90 | 34.75 | 27.00 | 58.75 | UGPR | KANP |
|  | Faizabad | E6 | 26.47 | 82.12 | 113.0 | 474.24 | 33.25 | 26.50 | 67.75 | MGPR | FAIZ |
|  | Ranchi | E7 | 23.17 | 85.19 | 625.0 | 1023.80 | 29.90 | 20.75 | 77.75 | EPH | RANC |
|  | Shillongani | E8 | 26.21 | 92.38 | 58.0 | 1013.14 | 30.25 | 23.50 | 84.25 | EHR | SHIL |
|  | Varanasi | E9 | 25.18 | 83.03 | 75.7 | 598.50 | 32.18 | 23.97 | 79.79 | MGPR | VARA |
|  | Mohanpur | E10 | 21.84 | 87.42 | 12.0 | 431.07 | 34.00 | 25.25 | 67.50 | LGPR | MOHA |
| North Western Plain Zone  (NWPZ) | Hisar | E11 | 29.10 | 75.46 | 215.2 | 214.30 | 34.95 | 25.71 | 75.15 | TGPR | HISA |
|  | Samba | E12 | 32.57 | 75.01 | 384.0 | 258.75 | 38.05 | 28.41 | 40.00 | WHR | SAMB |
|  | Mandore | E13 | 26.34 | 73.04 | 235.0 | 420.10 | 35.64 | 24.45 | 59.00 | WDR | MAND |
|  | Durgapura | E14 | 26.50 | 75.47 | 390.0 | 171.11 | 34.25 | 26.25 | 50.75 | WDR | DURG |
|  | Sriganganagar | E15 | 29.90 | 73.88 | 178.0 | 5.25 | 39.00 | 28.75 | 30.50 | TGPR | SRIG |
|  | New Delhi | E16 | 28.38 | 77.09 | 228.6 | 63.18 | 35.75 | 28.00 | 44.75 | TGPR | NEWD |
|  | Pantnagar | E17 | 29.00 | 79.30 | 243.8 | 546.78 | 29.00 | 21.50 | 71.75 | UGPR | PANT |
| Central Zone  (CZ) | Raipur | E18 | 21.16 | 81.36 | 289.6 | 775.50 | 33.50 | 18.50 | 70.75 | EPH | RAIP |
|  | SK Nagar | E19 | 24.15 | 72.02 | 154.52 | 480.2 | 34.2 | 24.1 | 65.0 | GPH | SKNA |
|  | Akola | E20 | 20.70 | 77.00 | 287.0 | 378.80 | 31.86 | 23.76 | 72.70 | WPH | AKOL |
|  | Navasari | E21 | 20.57 | 72.54 | 12.0 | 188.20 | 26.36 | 25.23 | 74.60 | GPH | NAVA |
|  | Jalgaon | E22 | 21.03 | 75.34 | 201.2 | 737.30 | 34.20 | 23.82 | 70.52 | WPH | JALG |
|  | Sagar | E23 | 24.27 | 78.21 | 530.0 | 105.30 | 30.14 | 22.75 | 71.00 | CPH | SAGA |
|  | Banda | E24 | 25.47 | 80.33 | 123.2 | 297.28 | 35.00 | 28.00 | 56.50 | CPH | BAND |
|  | Jagdalpur | E25 | 19.07 | 82.00 | 552.0 | 1593.40 | 30.36 | 22.11 | 82.57 | EPH | JAGD |
|  | Ambikapur | E26 | 23.13 | 83.18 | 623.0 | 698.44 | 30.80 | 22.60 | 71.60 | EPH | AMBI |
|  | Chitrakoot | E27 | 25.10 | 80.32 | 137.0 | 847.00 | 42.60 | 19.20 | 70.30 | MGPR | CHIT |
|  | Indore | E28 | 22.43 | 76.54 | 567.0 | 849.30 | 30.43 | 24.98 | 69.75 | CPH | INDO |
| South Zone  (SZ) | Dharwad | E29 | 14.15 | 75.20 | 678.0 | 376.2 | 26.8 | 21.2 | 64.2 | SPH | DHAR |
|  | Madhira | E30 | 16.15 | 80.22 | 189.0 | 543.40 | 33.41 | 23.62 | 68.20 | SPH | MADH |
|  | Warangal | E31 | 18.01 | 79.36 | 259.0 | 528.68 | 31.33 | 24.00 | 66.83 | ECPH | WARA |
|  | Vamban | E32 | 11.30 | 76.26 | 122.0 | 335.20 | 34.74 | 25.33 | 62.80 | SPH | VAMB |
|  | Coimbatore | E33 | 11.00 | 77.00 | 426.7 | 107.30 | 31.88 | 23.45 | 65.75 | SPH | COIM |
|  | Mandya | E34 | 12.52 | 76.89 | 678.0 | 482.85 | 29.00 | 20.75 | 75.75 | SPH | MAN |
|  | Aduthurai | E35 | 11.00 | 79.30 | 19.4 | 428.00 | 34.33 | 24.78 | 74.42 | ECPH | ADUT |
|  | Virinjipuram | E36 | 12.55 | 79.11 | 725.0 | 439.30 | 34.11 | 20.73 | 65.40 | SPH | VIRI |
|  | Berhampur(o) | E37 | 19.18 | 84.54 | 34.0 | 872.00 | 34.40 | 24.00 | 73.25 | EPH | BEHR |
|  | Keonjhar | E38 | 21.62 | 85.58 | 596.0 | 792.92 | 29.75 | 23.25 | 77.80 | ECPH | KEON |
|  | Banglore | E39 | 13.07 | 77.34 | 924.0 | 425.86 | 28.25 | 20.75 | 72.00 | SPH | BANG |

**AER; Agro–ecological regions, WHR; Western Himalayan Region, EHR; Eastern Himalayan Region,** MGPR; **Middle Gangetic Plain Region,** UGPR; **Upper Gangetic Plains Region, EPH; Eastern Plateau and Hills,** LGPR; **Lower Gangetic Plain Region,** TGPR; **Trans–Ganga Plains Region,** WDR; **Western Dry Region,** GPH; **Gujarat Plains and Hills,** WPH; **Western Plateau and Hills,** CPH; **Central Plateau and Hills,** SPH; **Southern Plateau and Hills,** ECPH; **Eastern Coastal Plains and Hills**

**Supplementary Table 2 ǀ**Detail information about the tested genotypes of mungbean over 39 locations

| S.N. | Genotype | Pedigree | Developing centre | State | DTF | DTM | 100–seed weight |
| --- | --- | --- | --- | --- | --- | --- | --- |
| 1 | AKM 12–24 | AKM 9911×AKM 9904 | PDKV, Akola | Maharashtra | 40 | 69 | 3.56 |
| 2 | AKM 12–28 | AKM 9911 ×BM 2003–2 | PDKV, Akola | Maharashtra | 40 | 68 | 3.58 |
| 3 | BM 2012–9 | Mutant of BPMR 145 | ARS, Badnapur | Maharashtra | 41 | 70 | 4.27 |
| 4 | COGG 13–39 | CO 6 ×SML 668 | TNAU, Coimbatore | Tamil Nadu | 43 | 71 | 3.64 |
| 5 | DGG 7 | Mutant of sel. 4 | ARS, Dharwad | Karnataka | 41 | 69 | 3.82 |
| 6 | IGKM 2016–1 | (Pairymung x Pusa vishal) | IGKV, Raipur | Chhattisgarh | 41 | 70 | 3.13 |
| 7 | IPM 410–9 | IPM 03–1 ×NM 1 | IIPR, Kanpur | Uttar Pradesh | 39 | 67 | 3.57 |
| 8 | IPM 512–1 | IPM 99–125 ×Co 5 | IIPR, Kanpur | Uttar Pradesh | 40 | 69 | 3.59 |
| 9 | JAUM 0936 | MH 96–1 ×SML 668 | SKUAST, Samba | Jammu & Kashmir | 40 | 69 | 3.57 |
| 10 | JLM 302–46 | BPMR–145 ×Local | MPKV, Jalgaon | Maharashtra | 40 | 68 | 3.46 |
| 11 | KM 2355 | KM 2241 ×KM 2273 | CSAUA&T, Kanpur | Uttar Pradesh | 40 | 68 | 3.19 |
| 12 | LGG 607 | MGG 295 ×P 109–5 | ARS, Lam | Andhra Pradesh | 47 | 74 | 3.34 |
| 13 | MDGGV–18 | Local sel. ×BPMR–145 | Mahodaya Hybrid Seeds Pvt. Ltd., Jalna | Maharashtra | 38 | 67 | 4.14 |
| 14 | MGG–387 | Madhiramung ×Asha–1–7 | ARS, Madhira | Telangana | 42 | 70 | 3.32 |
| 15 | MH 1323 | MH 318 x AKM 99–4 | CCSHAU, Hisar | Haryana | 38 | 67 | 3.69 |
| 16 | ML 2479 | Pusa 105 x ML 1354 | PAU, Ludhiana | Punjab | 42 | 70 | 3.38 |
| 17 | NDMK 16–324 | NDM 1 x MH 521 | NDUA&T, Faizabad | Uttar Pradesh | 40 | 69 | 3.33 |
| 18 | NMK 15–08 | Meha x GM 4 | NAU, Navsari | Gujarat | 41 | 69 | 3.90 |
| 19 | NVL–855 | NVS–242 (1) ×NVS–321s1 | Nirmal Seeds Pvt. Ltd., Pachora | Maharashtra | 40 | 68 | 4.32 |
| 20 | OBGG 56 | OBGG 52 × Kendrapara Local | OUAT, Berhampur | Odisha | 39 | 67 | 3.24 |
| 21 | OBGG 58 | VC 1560A ×VC 6370–92 | OUAT, Berhampur | Odisha | 42 | 69 | 3.71 |
| 22 | PM 14–11 | COGG 912 ×PM 5 | GBPUA&T, Pantnagar | Uttarakhand | 42 | 70 | 3.67 |
| 23 | PM 14–3 | PM 6 ×Pusa Ratna | GBPUA&T, Pantnagar | Uttarakhand | 41 | 69 | 3.77 |
| 24 | Pusa M 1771 | MH318×Pusa9531 | IARI, New Delhi | New Delhi | 40 | 67 | 3.78 |
| 25 | Pusa M 1772 | IPM02–14×Pusa vishal | IARI, New Delhi | New Delhi | 39 | 68 | 3.73 |
| 26 | RMB 12–07 | Pusa Vishal × DGG–1 | BAU, Ranchi | Jharkhand | 39 | 68 | 3.81 |
| 27 | RMG 1097 | RMG 492×MUM 2 | RARI, Durgapura | Rajasthan | 39 | 68 | 3.40 |
| 28 | SKNM 1502 | GM 9912 × GM 4 | SDAU, S.K. Nagar | Gujarat | 38 | 67 | 3.64 |
| 29 | SKNM 1504 | GM 9923 × GM 3 | SDAU, S.K. Nagar | Gujarat | 38 | 68 | 3.75 |
| 30 | SML 1808 | ML 1349 × Mash 1–1 | PAU, Ludhiana | Punjab | 39 | 68 | 3.66 |
| 31 | SVM–6133 | SML–668 × Pusa–9531 | SVHS, Hisar | Haryana | 39 | 67 | 4.40 |
| 32 | TMB 126 | Samrat × Kopergaon | BARC, Mumbai | Maharashtra | 40 | 68 | 3.94 |
| 33 | VGG 16–036 | IPM 03–01 × SPS 5 | NPRC, Vamban | Tamil Nadu | 44 | 72 | 3.36 |
| 34 | VGG 16–055 | VBN(Gg)2×SM47 | NPRC, Vamban | Tamil Nadu | 40 | 68 | 3.69 |

DTF, days to 50% flowering; DTM, days to maturity;
